# Retention, mobility, and successful transition to independence of health sciences postdocs

**DOI:** 10.1371/journal.pone.0276389

**Published:** 2022-11-01

**Authors:** C. Elizabeth Shaaban, Tammy L. Dennis, Stephen Gabrielson, Laura J. Miller, Darlene F. Zellers, Arthur S. Levine, Caterina Rosano

**Affiliations:** 1 Department of Epidemiology, School of Public Health, University of Pittsburgh, Pittsburgh, PA, United States of America; 2 Office of Academic Career Development, Health Sciences, University of Pittsburgh, Pittsburgh, PA, United States of America; 3 Health Sciences Library System, University of Pittsburgh, Pittsburgh, PA, United States of America; 4 Brain Institute, University of Pittsburgh, Pittsburgh, PA, United States of America; Rutgers University, UNITED STATES

## Abstract

**Introduction:**

Obtaining a tenure track faculty position (TTFP) after postdoctoral appointment (PDA) completion is considered an indicator of successful transition to independence (TTI). Whether cross-institutional mobility (CIM)—moving to a different institution from that of the PDA—contributes to TTI is unclear, as data evaluating retention and mobility is lacking. We tested the hypothesis that, for postdocs (PDs) at R1 institutions, CIM is a significant predictor of successful TTI defined as TTFP-status 3 years post-PDA.

**Materials and methods:**

Using University of Pittsburgh data for health sciences PDs we tested the association of CIM at PDA completion (moved to a different institution (CIM = 1) or retained at Pitt (CIM = 0)) with TTFP-status 3 years post-PDA (TTFP, non-TTFP, or left faculty position) using multinomial logistic regression models.

**Results:**

Among all 622 Pitt PDs, 3-year retention in a faculty position at Pitt was 21%, while 14% had a faculty position outside of Pitt. Among the analytic sample of PDs with an academic career outcome during the study period (N = 238; 50% women, 8% underrepresented minorities (URM)), at baseline PDA completion 39% moved to a different institution (CIM = 1), and 61% remained at Pitt (CIM = 0) in any job type. Those with CIM = 1 had greater odds of having a TTFP at follow-up than those with CIM = 0 [adjusted OR (95% CI): 4.4 (2.1, 9.2)].

**Discussion:**

One fifth of Pitt PDs were retained by Pitt as faculty. While Pitt PDs were equally likely to get a faculty position whether they were retained at Pitt or left, those who left had greater odds of obtaining a TTFP. Future work with longer follow-up times, expanded markers of TTI, and samples from other R1 institutions is needed to better understand the reason for these results. This knowledge can lead to better support for the next generation of PDs as they successfully transition to faculty.

## Introduction

Transitioning to a tenure track faculty position (TTFP) after completion of a postdoctoral appointment (PDA) is an early marker of reaching scientific independence, a major goal of academic scientists and the focus of National Institutes of Health (NIH) T32 training programs and other non-T32 mentoring activities [2–4]. Nearly $700 million in 2020 National Institutes of Health funding (T32 and F32 funding combined) [5] and innumerable hours are invested in training activities to promote successful transition to independence (TTI) for postdocs (PDs). A rigorous assessment of the efficacy of activities and initiatives to support TTI is critical to improve our training programs and support the success of the next generations of faculty. Several qualitative analyses have examined predictors of TTI; however, systematic assessments are limited and there is little objective information of the actual impact of specific factors that promote successful TTI.

Several reports describing predictors of TTI have identified personal factors including one’s own autonomy and initiative, networks, ability to persevere through challenges, and work life balance as well as organizational factors including access to appropriate institutional support and resources, having protected time to focus on research, and receipt of good mentorship [4,6,7]. Increasing academic diversity and equity has become a key goal of many institutions, and guidance specifically directed at underrepresented minorities (URM) has been provided focusing on many of these same factors [8]. Other TTI-related factors include the ability to pursue novel directions and to obtain preliminary data to secure future funding [2,3]. However, for the most part, these predictors are difficult to measure objectively.

Unlike the other predictors of TTI mentioned above, cross-institutional mobility (CIM) is easily quantifiable and measurable, however little research assessing retention and CIM has been reported. For example, according to published survey results, CIM was commonly reported by new UK-based principal investigators [9]. The majority of respondents endorsed having moved at least once over the course of their academic careers, but career stage was not delineated, and the relationship of CIM with TTI was not evaluated [9]. In another study at an R1 university (a designation from the Carnegie Classification of Institutions of Higher Education indicating doctoral degree granting universities with very high research activity [1]), researchers at the University of California, San Francisco (UCSF) reported that 37% (N = 336/899) of non-MD biomedical, behavioral, and clinical research PDs obtained faculty or “faculty-like” positions—research or teaching positions at academic, research, or government institutions. UCSF retained about 10% (N = 92/899) as faculty, and of this group, 21% (N = 19/92) were in TTFPs. Thus, most of the PDs (27%, N = 244/899) moved to a new institution for their faculty position. However, the capture of the data as “faculty-like” positions at research and government institutions in addition to academic faculty positions did not allow differentiation of TTFP vs. non-TTFP positions among this externally-placed sub-group and across the full study sample [10]. Our objectives in this study were to assess the proportion of health sciences PDs retained in faculty positions at our institution vs. those who leave and to test the hypothesis that, for PDs coming from R1 universities such as our institution, CIM is a predictor of successful TTI defined as having a TTFP 3-years post-PDA.

## Materials and methods

### Study design

**Fig 1:**
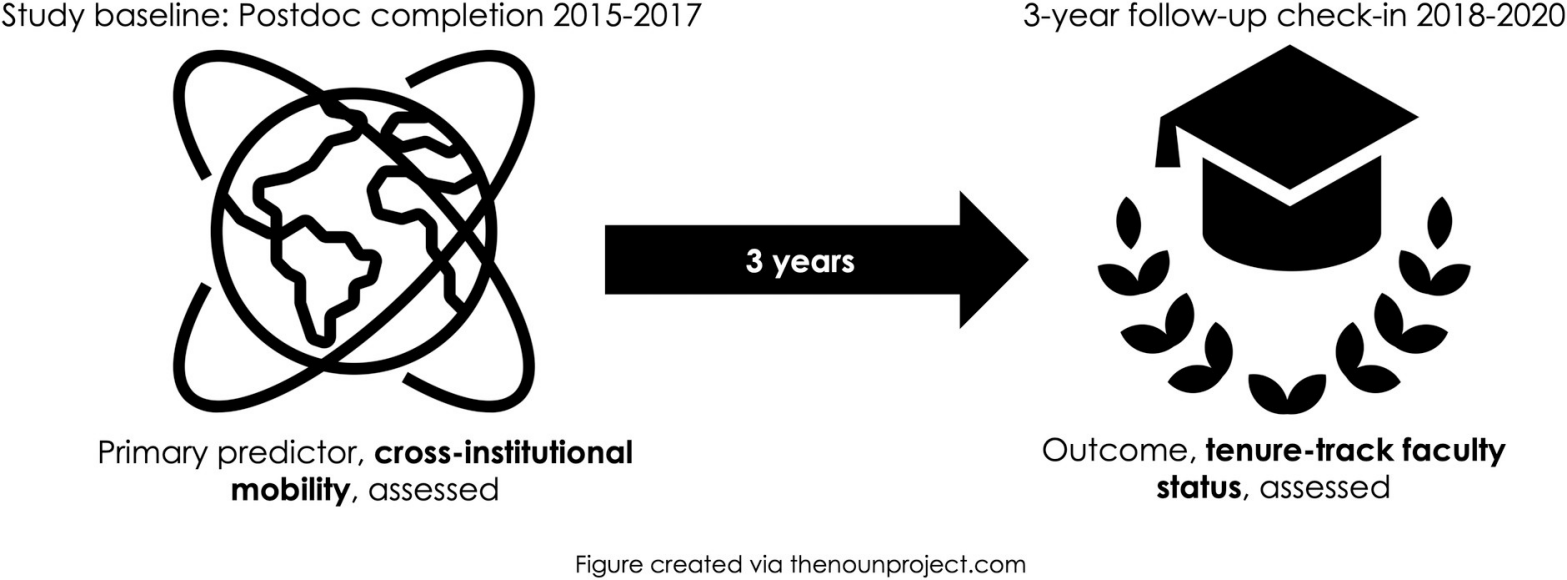
Study design.

The study design is visualized in Fig 1. The primary predictor, CIM, was assessed at PDA completion in 2015–2017 (study baseline). TTFP-status follow-up data was collected via internet searches of publicly available profiles and websites during a check-in from 2018–2020, 3 years after PDA completion.

### Participants

We obtained data previously collected by the Office of Academic Career Development, Health Sciences, at the University of Pittsburgh (Pitt) as part of their efforts to track 3-year career outcomes among all PDs from the following schools: Dental Medicine, Health and Rehabilitation Sciences, Medicine, Nursing, Pharmacy, and Public Health. Classification of the workforce sector, job type, and location using our unique 5-tier career outcomes taxonomy was available for all PDs who completed their PD appointment (PDA) from 2015–2017, the baseline of this study, and at 3 years post-PDA completion 2018–2020 (N = 622).

This study was reviewed and approved as an expedited study with a waiver of informed consent by the University of Pittsburgh Institutional Review Board (STUDY21070001). Participant confidentiality and analyst objectivity were further protected by providing only deidentified data to investigators / analysts outside of the Pitt Office of Academic Career Development. This office already has access to identifiable information due to the nature of its work tracking PD training and outcomes.

Deidentified data used in these analyses is available on the Open Science Framework: https://doi.org/10.17605/OSF.IO/7XB4D.

### Predictor

CIM was classified as having obtained a baseline position outside of Pitt (CIM = 1) or being retained for a baseline position at Pitt (CIM = 0). MDs (N = 11) whose baseline position was to complete their clinical residency rotation with Pitt’s affiliated teaching hospital system were classified as CIM = 0.

### Outcome

The operational definition of TTI in this study was TTFP-status at 3 years after PDA completion. TTFP-status was coded as “TTFP”, “non-TTFP”, or “left faculty position”. If not specifically known, tenure track and non-tenure track status was based on faculty titles, a practice used by Academic Analytics [11], a research organization providing analytic services to higher education institutions. For example, “Research Assistant Professor” would be coded as non-tenure track whereas “Assistant Professor” would be coded as tenure track when the title-based approach to coding is used.

### Covariates

We also collected data on gender, underrepresented minority (URM) status (comprised of individuals who reported American Indian or Alaska Native, Black or African American, or Hispanic / Latino (of any race) race/ethnicity), US citizen / permanent resident status, length of PDA in months, and whether PDs had a faculty position at PDA completion (baseline).

### Statistical analyses

We present descriptive statistics as median (25^th^ %ile, 75^th^ %ile) tested with the Wilcoxon-Mann-Whitney test or N (%) tested with Fisher’s exact test. We tested the hypothesis that CIM at PDA completion is a significant predictor of TTFP-status at 3 years post-PDA (our measure of TTI) using multinomial logistic regression modeling; non-TTFP was set as the reference category. Results are presented as Odds Ratios (OR) and their 95% confidence intervals (CI) for an unadjusted Model 1, a model adjusted for gender and underrepresented minority (URM) status (Model 2), and a model additionally adjusted for US citizen / permanent resident status, length of PDA, and faculty position at baseline (Model 3). We tested interactions of gender and URM status with CIM and included them if p<0.10.

Some PDs had 3-year check-ins in 2020 including the time during the COVID-19 pandemic (N = 76). To test whether our results were robust to inclusion/exclusion of data potentially impacted by the pandemic, we carried out a sensitivity analysis repeating Fisher’s exact testing and multinomial logistic regression modeling withholding PDs with 2020 check-ins from the analysis.

Alpha was set at 0.05. All analyses were carried out in SAS version 9.4 [12].

## Results

Among the 622 PDs at the University of Pittsburgh, a total of 267 (42.9%) had an academic career workforce sector and faculty job type during the study period (at baseline or 3-year follow-up) and 355 (57.1%) did not. Overall faculty placement at the year 3 follow-up was 217/622 (34.9%): 132/622 (21.2%) had a faculty position at Pitt, while 85/622 (13.7%) had a faculty position outside of Pitt. Overall TTFP placement was 147/622 (23.6%; raw numbers in Table 1).

**Table 1:** Postdoc characteristics.

	All N = 238		CIM = 1 (Left Pitt) at baseline N = 93 (39.1%)		CIM = 0 (Retained at Pitt) at baseline N = 145 (60.9%)		p
Baseline Characteristics							
Women	119	50.0%	52	55.9%	67	46.2%	0.18
Underrepresented minority	18	7.6%	9	9.7%	9	6.2%	0.33
Nationality: US Citizens & Permanent Residents	187	78.6%	74	79.6%	113	77.9%	0.87
Months in postdoc, median (Q1, Q3)	30	23, 40	24	16, 36	34	24, 47	0.006
Had a faculty position at baseline	195	81.9%	76	81.7%	119	82.1%	>0.99
Faculty position status at 3-year follow-up							< .0001*
Left baseline faculty position	21	8.8%	8	8.6%	13	9.0%	
Total with a faculty position at 3-year follow-up	217	89.5%	85	91.4%	132	91.0%	
Tenure track position	147	61.8%	72	77.4%	75	51.7%	
Non-tenure track position	70	29.4%	13	14.0%	57	39.3%	

Statistics are N % unless otherwise noted. CIM = cross-institutional mobility; Q = quartile. *p-value is from a Fisher’s exact test comparing proportions with tenure-track faculty positions, non-tenure track faculty positions, and who left a baseline faculty position by the 3-year follow-up.

**Fig 2:**
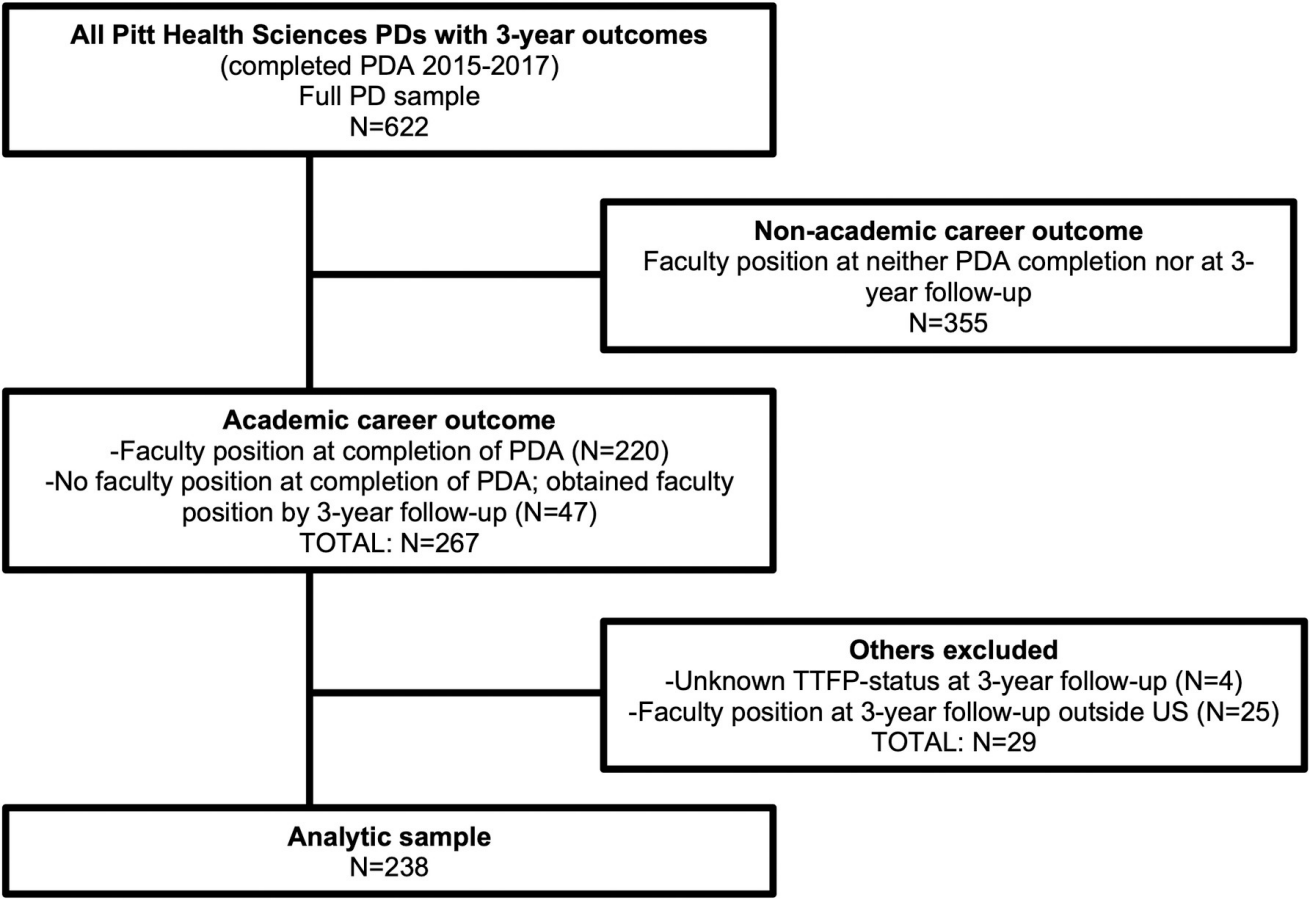
Inclusion of analytic sample. Note: PD = postdoc; PDA = postdoctoral appointment; TTFP = tenure-track faculty position.

Most of the 267 with academic career outcomes obtained their faculty position at baseline, immediately upon PDA completion (N = 220 (82.4%)), with the remaining (N = 47 (17.6%)) not having a faculty position at baseline but obtaining one by the 3-year follow-up. Among the 267, N = 4 had an unknown TTFP-status at the 3-year follow-up, and N = 25 had obtained a faculty position outside of the US and were removed due to the lack of a comparable tenure system. This left a total of N = 238 for our analytic sample of TTI predictors (Fig 2). Information about the 355/622 who had non-academic career workforce sector positions or worked in the academic career workforce sector in a non-faculty role, revealed these individuals went on to jobs with non-profits, industry, or staff positions at academic institutions; we did not review their data any further.

### Analytic sample (N = 238) at baseline

Among the analytic sample (50.0% women, 7.6% URM, 78.6% US citizen or permanent resident; Table 1), more PDs were retained at Pitt than left Pitt, N = 145 (60.9%) vs. N = 93 (39.1%) (Table 1). There was a significant association between CIM and length of the PDA, but not gender, URM status, US citizen/permanent resident status, or having a faculty position at PDA completion (Table 1). Those with CIM = 1 had a shorter median PDA by nearly one year than those with CIM = 0 (p = 0.006; Table 1).

### Analytic sample at 3-year follow-up

Overall, 61.8% of PDs had a TTFP, 29.4% had a non-TTFP, and 8.8% had left a faculty position they previously held (Table 2). Equal proportions of those who were retained by Pitt and left Pitt had a faculty position at the 3-year follow-up (91%, Table 1). In raw numbers, an equal number of PDs who were retained by Pitt and left Pitt achieved a TTFP (CIM = 0: N = 75; CIM = 1: N = 72; Table 1). However, the proportions differed (51.7% vs. 77.4%), and in the overall Fisher’s exact test, CIM was associated with TTFP-status (Table 1). More specifically in pairwise comparisons, leaving a faculty position was not associated with CIM (p>0.99), while both TTFP and non-TTFP were (both p’s < .0001). In multinomial logistic regression models, there was a significant positive association between CIM and TTFP at 3 years—those who moved to a new institution had 4.2 times the odds of having a TTFP at 3 years, compared to those who did not move to a new institution (Table 2). Those with CIM = 1 also had 2.7 times the odds of leaving faculty vs those with CIM = 0, though this association was non-significant (Table 2). Adjusting for gender and URM status did not alter these results (Model 2, Table 2). Interactions of CIM with gender and URM status had p>0.50 and were not included in the final models. Further adjustment for US citizen / permanent resident status, length of PDA, and baseline faculty position did not appreciably change the association of CIM with TTFP (adjusted OR: 4.4, Model 3, Table 2).

**Table 2:** Association of cross-institutional mobility (CIM) at baseline with tenure track faculty position status at 3-year follow-up among 238 PDs.

Multinomial logistic regression			
	Odds Ratio	95% Confidence Interval	
		Lower	Upper
**Model 1: Unadjusted**			
Tenure track	4.2	2.1	8.3
Left faculty	2.7	0.9	7.8
**Model 2: Adjusted for gender, underrepresented minority status**			
Tenure track	4.1	2.1	8.2
Left faculty	2.8	0.9	8.1
**Model 3: Model 2 + further adjusted for US citizen / permanent resident status, length of postdoctoral associate appointment, faculty position at baseline**			
Tenure track	4.4	2.1	9.2
Left faculty	2.8	0.9	8.5

Note: Non-tenure track faculty position is the reference category.

Sensitivity analyses withholding PDs with 3-year check-ins during the COVID-19 pandemic (2020) showed that proportions of TTFP-status at follow-up by CIM were similar to those in the full sample and statistically different (p<0.0001, S1 Table). Multinomial logistic regression results were stronger than in the full sample, although with wider confidence intervals due to the smaller sample size (S2 Table). In these sensitivity analyses, those who moved to another institution vs. those who remained at Pitt at baseline were significantly more likely to have left their faculty position by follow-up vs. being in a non-TTFP position (S2 Table).

## Discussion

Our study provides some of the first available estimates of retention rates of PDs in faculty positions and associations between CIM and TTFP among those completing PDAs at R1 institutions. Our first key result is that about 21% of all Pitt PDs in our study were retained by Pitt in faculty positions, double the retention rate reported among PD alumni at UCSF (10%) [10], and among the analytic sample of Pitt health sciences PDs with an academic career outcome, more than half were retained by Pitt at PDA completion. By 3 years post-PDA, 52% of these were in a TTFP, also more than double the proportion found at UCSF (21%) [10]. When considering comparisons of these rates, it is important to note that the Pitt and UCSF samples differed in several ways. Our samples varied on 1) inclusion of type of doctoral degree (our sample included MDs while the UCSF study did not); 2) sources of health-related PDs (we included all PDs completing from 2015–2017 from any of our 6 schools of health sciences while the UCSF study included PDs working in NIH T32 training grant supported labs who themselves had been supported by any funding source); 3) follow-up time (our study looked at 3-year outcomes while the UCSF study looked across 13-year outcomes); and 4) workforce sector (we included only faculty positions in the academic workforce sector while the UCSF study included faculty or “faculty-like” positions at academic, research, and government institutions) [10]. Despite these differences, the comparison is reasonable as both universities are R1 institutions. Benchmarking retention estimates is difficult because few institutions have publicly reported this metric. Because the literature on retention is limited, we recommend that retention rates of PDs as faculty overall and by TTFP-status at other academic institutions be reported.

Our second key result is that moving to a different institution was significantly associated with greater odds of being in a tenure track faculty position after 3 years, and this association was not explained by other factors measured. This result was also not due to variations in faculty job-seeking and hiring patterns seen during the COVID pandemic. Thus, while Pitt retains more PDs as faculty and as TTFP than another R1 institution has reported, the PDs who were retained at PDA completion in our study were less likely to obtain a later TTFP than those who were mobile. There are several possible explanations for this association. One is a causal pathway in which exposure to numerous perspectives and disciplinary and training styles obtained at differing institutions may enhance successful TTI; if this is the case, recommending CIM to enhance PDs’ TTI would be sensible. Another possible explanation for this association is reverse causality in which a TTFP offer causes CIM. For example, PDs may be unlikely to leave the institution where they completed their PDA unless they have a TTFP offer. This would cause those with a non-TTFP offer at their PD institution to stay in place while those with TTFP offers would move. We addressed this issue by using a 3-year time separation between our predictor, CIM, and our outcome, TTFP-status 3-years post-PDA completion, and adjusting for baseline faculty job status. Nevertheless, 3 years may not be a long enough separation, and reverse causality may still partially explain the relationship we observe. Five-year post-PDA completion check-ins are planned as part of our future work, and this longer-term follow-up data can help to further clarify this pathway. If this pathway is correct, other recommendations to enhance TTI would need to be identified because promoting CIM would not cause improved TTI as measured by TTFP. There could instead be a pathway in which the association of CIM with TTI is spurious due to confounding by academic norms and reviewer expectations irrespective of PD caliber—beliefs are held by appointment, tenure, and promotion committees and grant and manuscript reviewers that lead both to mobility and success. If it is reviewer expectations of CIM that bias which PDs can be successful, recommendations should center around altering reviewers’ expectations for CIM. Our future work will attempt to further tease this apart by evaluating multiple additional metrics of TTI including publication metrics in which CIM is less likely to be part of the review process than for TTFPs where CIM is likelier to be a central part of the review process. These results may also be due to confounding by other unmeasured factors, and if this is the case, again intervening on CIM would not promote TTI and other recommendations will be needed. In addition, these results may be less applicable to R2 universities or R1 universities with few non-TTFP positions available; they are most generalizable to PDs from other similar, R1 universities offering both TTFP and non-TTFP. Further, these are results from one institution, and patterns of retaining PDs as faculty at the same institution may be influenced by departmental or institutional culture. There may be something unique about Pitt retaining PDs and evaluating this association among PDs at other R1 universities can help expand generalizability and clarify the best recommendations to promote TTI under varying circumstances.

Other considerations regarding faculty placement are noteworthy. We found that among our full health sciences PD sample, 35% had a faculty position 3 years post-PDA completion, similar to the 37% 13-year placement rate seen at UCSF [10], and nearly one quarter of the PDs in our study had a TTFP placement. The TTFP estimate is in line with estimates from both the 2012 NIH Biomedical Workforce Working Group Report on the proportion of biomedical PhD-holders with a TTFP (23%) across a 15-year period, and a report of National Institute of Environmental Health Sciences within NIH of PD alumni (30%) across a 14-year period [13,14]. The consistency with our results is all the more remarkable considering that follow-up in these reports was longer compared to ours (3 years). Although their analysis is not specific to PDs, the NIH Biomedical Workforce Report shows that the proportion of PhD holders with a TTFP increases from 1–5 years post PhD to 6+ years post PhD [14].

Our results do not necessarily imply that those retained in non-TTFP are less independent than those in TTFP when other measures of TTI (e.g., grants awarded, publication impact) are considered, and our future work will further investigate this. The results from our study provide an important scaffolding upon which future research in this area can be built. The difficulty in clarifying the explanatory pathways operating in TTI point out that more work is needed in this area, but progress in this field has been limited by the lack of a clear definition of TTI. Traditional definitions have focused on being in a TTFP and self-sufficient with funding (e.g., receipt of first R01). Newer definitions have also been proposed including independence of thought—the freedom to choose scientific questions of interest and how to approach them [2]. Measuring independence as the time to first R01 award creates several analytical challenges, because it takes several years from time of first appointment as faculty to time of obtaining an R01 award; data recently published by NIH suggest that age at first R01 has increased from a mean age of 40 in 1995 to a mean age of 44 in 2020 [15]. Other markers of TTI include number and impact of publications [16] and scientific good citizenship [3], which includes providing peer review of the scientific literature and grants, leadership in national professional organizations, and mentorship of earlier career trainees. However, these data are not routinely collected and difficult to quantify objectively for several reasons. Authors may publish under multiple names, have the same name as other authors, or have name indexing variations especially in the case of varying name conventions across countries of origin. Also, information about grant and manuscript reviews, professional leadership positions, and mentorship are not centrally collected in the same manner as publications. They thus require a large investment of work to research and collect accurately. Moreover, absolute number of publications may not accurately reflect TTI; for example, publications with one’s mentor may reflect less independence than publications with different investigators. We also acknowledge that the very metrics most often used to track TTI may not reflect the full range of what academics do and value such as disseminating work to the public and influencing policy [17–19]. This has important equity implications, disproportionately advantaging some scholars while disproportionately disadvantaging others. Altmetrics can provide additional measures of scholarly impact such as policy influence and popular press, blog, and social media engagement with research [20]. Initiatives like the HuMetricsHSS Humane Metrics Initiative [21] are attempting to develop measures of impact reflecting the full range of activities that scholars value, and these types of metrics will be important to incorporate into this body of research moving forward [22]. Additional guidance regarding responsible assessment of research impact and use of metrics has been provided through the San Francisco Declaration on Research Assessment [23] and the Leiden Manifesto for research metrics [24].

We note that a small proportion of PDs in our study were URM, although it was greater than the proportion among 2019 US PDs in science, engineering, and health (7.6% in our study vs. 4.6% nationally [25]). Overall these proportions are far below the proportion of URM in the US population (33.2%) [26]. As research on TTI progresses, it is important to keep considerations of equity in the forefront. Of specific relevance to CIM, mobility may counter important institutional and societal goals relating to equity in the academy. New approaches to prioritizing equity in the academy have been developed which prevent CIM such as PD to TTFP conversion programs [27]. These programs are designed to retain PDs from URM backgrounds as tenure track faculty. CIM can also adversely impact PDs who need to prioritize family and financial concerns, and these are known to be important deciding factors when PDs, especially women and URM PDs, determine whether to remain on the academic career path [28]. Therefore, we caution against maintaining academic norms that require mobility without thinking critically about all possible consequences, including purposefully considering potential negative consequences.

In addition to those already mentioned earlier in the discussion, several strengths and limitations should be kept in mind when considering these results. Key strengths of this work are that it 1) uses objective data vs. subjective, self-reported data (such as survey data); 2) is an analysis of a full sample of all health sciences PDs at an institution over a defined period rather than a convenience sample which is subject to bias in who volunteers to take part; and 3) is longitudinal, with baseline data at PDA completion and follow-up after 3 years. Limitations include that we do not have information on PD career preference, and therefore we were unable to evaluate faculty outcomes among PDs who may have desired a faculty position but were unable to obtain one, making these results subject to survivorship bias. In addition, there are many reasons a candidate may choose to accept or decline a job offer rather than simply prioritizing TTFP offers. Given the nature of our study, we do not have information on the factors candidates weight when choosing to accept or decline an offer. Future studies using survey, interview, or focus group designs would be well-positioned to collect such data. Another key limitation is that we do not have information about prior PDAs at other institutions; PDs with prior PDAs may have had more time to publish, win grants, build networks, and learn new skills, all of which may be predictive of TTI. Furthermore, having this data would allow us to evaluate the association of CIM at different career stages with later TTFP. Our research is ongoing, and we are presently collecting 3-year outcomes on additional cohorts of completing PDs as well as 5-year outcomes.

## Conclusions

We provide some of the earliest available estimates of retention rates of PDs in faculty and TTFP positions at an R1 university. While having a faculty position was equally likely for those who were retained vs. those who were mobile, the likelihood of TTFP vs. non-TTFP was higher for those who were mobile. Longer follow-up times with expanded markers of TTI and studies at other institutions are needed to better assess both the reason for the association of CIM with TTI and TTI more broadly. Our own future work will seek to better understand whether those who are mobile (vs. being retained by the PD institution) have more impactful and independent research work based on additional metrics of TTI. This knowledge can lead to better support for the next generation of PDs as they successfully transition to faculty.

## Supporting information

S1 Table Faculty position status at 3-year follow-up among postdocs without check-ins in 2020. Statistics are N %. CIM = cross-institutional mobility. *p-value is from a Fisher’s exact test comparing proportions with tenure-track faculty positions, non-tenure track faculty positions, and who left a baseline faculty position by the 3-year follow-up.
(DOCX)

S2 Table Association of cross-institutional mobility (CIM) at baseline with tenure track faculty position status at 3-year follow-up among 162 PDs without check-ins in 2020. Note: Non-tenure track faculty position is the reference category.
(DOCX)

## Abbreviations

Academic career: a faculty position in an academic institution
CIM: cross-institutional mobility; moving to a new institution for the next stage of a career. Moved to a new institution (CIM = 1) vs. retained at the same institution (CIM = 0)
PD: postdoc; an academic role focused on additional training open to people who have obtained a doctorate degree
PDA: postdoctoral appointment; the postdoc job contract for a defined period of time
R1: a designation from the Carnegie Classification of Institutions of Higher Education indicating doctoral degree granting universities with very high research activity [: 1: ])
TTFP-status: tenure-track faculty position-status; used as the outcome in this study at the 3-year follow-up check in and coded as tenure-track position (TTFP), non-tenure track position (non-TTFP), or left faculty position
TTI: transition to independence; operationally defined as moving from a postdoc position to a TTFP
