# Supplementary material for: Retention, mobility, and successful transition to independence of health sciences postdocs
Source: PLoS One. 2022 Nov 1;17(11):e0276389. doi: 10.1371/journal.pone.0276389 (PMC9624420; doi:10.1371/journal.pone.0276389)
Supplement: S1 Table — Statistics are N %. CIM = cross-institutional mobility. *p-value is from a Fisher’s exact test comparing proportions with tenure-track faculty positions, non-tenure track faculty positions, and who left a baseline faculty position by the 3-year follow-up. (DOCX) [file pone.0276389.s001.docx]

**S1 Table. Faculty position status at 3-year follow-up among postdocs without check-ins in 2020.**

|  | **All**  **N=162** | | **CIM=1 (Left Pitt)**  **at baseline**  **N=67 (41.4%)** | | **CIM=0 (Retained at Pitt) at baseline**  **N=95 (58.6%)** | | **p** |
| --- | --- | --- | --- | --- | --- | --- | --- |
| Left baseline faculty position | 11 | 6.8% | 6 | 9.0% | 5 | 5.3% | <.0001* |
| Total with a faculty position at 3-year follow-up | 151 | 93.2 | 61 | 91.0% | 90 | 94.7% |  |
| Tenure track position | 103 | 63.6% | 54 | 80.6% | 49 | 51.6% |  |
| Non-tenure track position | 48 | 29.6% | 7 | 10.5% | 41 | 43.2% |  |

Statistics are N %. CIM=cross-institutional mobility. *p-value is from a Fisher’s exact test comparing proportions with tenure-track faculty positions, non-tenure track faculty positions, and who left a baseline faculty position by the 3-year follow-up.
