# Supplementary material for: Retention, mobility, and successful transition to independence of health sciences postdocs
Source: PLoS One. 2022 Nov 1;17(11):e0276389. doi: 10.1371/journal.pone.0276389 (PMC9624420; doi:10.1371/journal.pone.0276389)
Supplement: S2 Table — Note: Non-tenure track faculty position is the reference category. (DOCX) [file pone.0276389.s002.docx]

**S2 Table. Association of cross-institutional mobility (CIM) at baseline with tenure track faculty position status at 3-year follow-up among 162 PDs without check-ins in 2020.**

| **Multinomial logistic regression** | | | |
| --- | --- | --- | --- |
|  | Odds Ratio | 95% Confidence Interval | |
|  |  | Lower | Upper |
| **Model 1: Unadjusted** | | | |
| Tenure track | 6.5 | 2.7 | 15.7 |
| Left faculty | 7.0 | 1.7 | 29.4 |
| **Model 2: Adjusted for gender, underrepresented minority status** | | | |
| Tenure track | 6.2 | 2.5 | 15.3 |
| Left faculty | 7.2 | 1.7 | 31.0 |
| **Model 3: Model 2 + further adjusted for US citizen / permanent resident status, length of postdoctoral associate appointment, faculty position at baseline** | | | |
| Tenure track | 6.3 | 2.3 | 17.1 |
| Left faculty | 9.9 | 2.1 | 47.8 |

Note: Non-tenure track faculty position is the reference category.
